# Screening and Identifying Immune-Related Cells and Genes in the Tumor Microenvironment of Bladder Urothelial Carcinoma: Based on TCGA Database and Bioinformatics

**DOI:** 10.3389/fonc.2019.01533

**Published:** 2020-01-15

**Authors:** Jinlong Cao, Xin Yang, Jianpeng Li, Hao Wu, Pan Li, Zhiqiang Yao, Zhichun Dong, Junqiang Tian

**Affiliations:** ^1^Department of Urology, The Second Hospital of Lanzhou University, Lanzhou, China; ^2^Key Laboratory of Urological Diseases of Gansu Provincial, Lanzhou, China; ^3^Reproductive Medicine Center, The Second Hospital of Lanzhou University, Lanzhou, China

**Keywords:** bladder urinary cancer, immunotherapy, tumor microenvironment, TCGA, bioinformatics

## Abstract

Bladder cancer is the most common cancer of the urinary system and its treatment has scarcely progressed for nearly 30 years. Advances in checkpoint inhibitor research have seemingly provided a new approach for treatment. However, there have been issues predicting immunotherapeutic biomarkers and identifying new therapeutic targets. We downloaded the gene expression profile and clinical data of 408 cases bladder urinary cancer from the Cancer Genome Atlas (TCGA) portal, and the abundance ratio of immune cells for each sample was obtained via the “Cell Type Identification by Estimating Relative Subsets of RNA Transcripts (CIBERSORT)” algorithm. Then, four survival-related immune cells were obtained via Kaplan-Meier survival analysis, and 933 immune-related genes were obtained via a variance analysis. Enrichment, protein-protein interaction, and co-expression analyses were performed for these genes. Lastly, 4 survival-related immune cells and 24 hub genes were identified, four of which were related to overall survival. More importantly, these immune cells and genes were closely related to the clinical features. These cells and genes may have research value and clinical application in bladder cancer immunotherapy. Our study not only provides cell and gene targets for bladder cancer immunotherapy, but also provides new ideas for researchers to explore the immunotherapy of various tumors.

## Introduction

Bladder cancer is the nineth most common malignant neoplasm in the world. It is mainly represented by bladder urothelial carcinoma (BUC), which accounts for >90% of bladder cancer, and smoking is recognized as the most common risk factor (1, 2). The traditional treatments for bladder cancer mainly include surgical resection and chemotherapy, but there is a high recurrence rate, and the 5 year overall survival rate remains at 15–20% (3, 4). Owing to advancements in immuno-oncology and the introduction of checkpoint inhibitors in clinical practice for many cancers in recent years, there is hope for progress in the treatment of bladder cancer (5). Bacillus Calmette–Guérin is considered the earliest immunotherapeutic drug applied to bladder cancer, but its clinical application is limited due to low efficiency and high toxicity. Immunological checkpoint inhibitors are immunotherapeutic drugs that have emerged in recent years, and many clinical trials on these drugs are proceeding (6). A study from the Cancer Genome Atlas (TCGA) showed that bladder cancer is a disease with a large number of different genetic mutations, and may be sensitive to immunotherapy due to the high number of identifiable antigens (7). This finding suggests that immunotherapy may be beneficial in the treatment of bladder cancer. Many clinical trials of bladder cancer immunotherapy are currently in progress, but there are no results on efficacy, particularly compared to traditional cisplatin-based chemotherapy. Moreover, no approval has been given to any form of bladder cancer immunotherapy according to the Food and Drug Administration (6). The beneficiaries of immunotherapy are still limited to small-scale populations, and tumor-induced immune escape is a ubiquitous phenomenon. Many problems remain to be solved in BUC immunotherapy, especially in the field of predicting immunotherapeutic biomarkers and identifying new effective therapeutic targets.

Studies on the tumor microenvironment have been increasingly published in the field of cancer immunotherapy (8). The tumor microenvironment is the surrounding environment in which tumor cells reside, and consists of immune cells, mesenchymal cells, endothelial cells, inflammatory mediators, and extracellular molecules (9, 10). It is a powerful protective net for tumor cells formed in the fight between the tumor and the immune system, and it is also the premise and guarantee of tumor immune escape. Immune components in the tumor microenvironment have essential effects on gene expression by tumor tissues and the clinical outcome (11–13).

Cancer immunotherapy mainly works with some important proteins to enhance function or restore immune cells in the tumor microenvironment. Thus, we first explored survival-related immune cells in BUC, and then explored genes that are critical to the level of immune cell infiltration. In this study, the RNA-sequencing (RNA-Seq) gene expression profile and clinical data of 408 patients with BUC were downloaded from the TCGA database, and data extraction and analysis were performed with R software. A total of four survival-related immune cells and 24 hub genes were identified, four of which were related to survival. We validated the immune correlation through the online website “Tumor Immune Estimation Resource (TIMER).” Our study provides ideas and clues to BUC immunotherapy, and the cells and genes that were identified could be considered biomarkers for prognosis or targets for BUC therapy. Furthermore, this study also provides a new approach for immunotherapy researchers to explore immunotherapeutic cells and gene targets for the first time.

## Materials and Methods

### Data Source and Pre-processing

**Figure 1:**
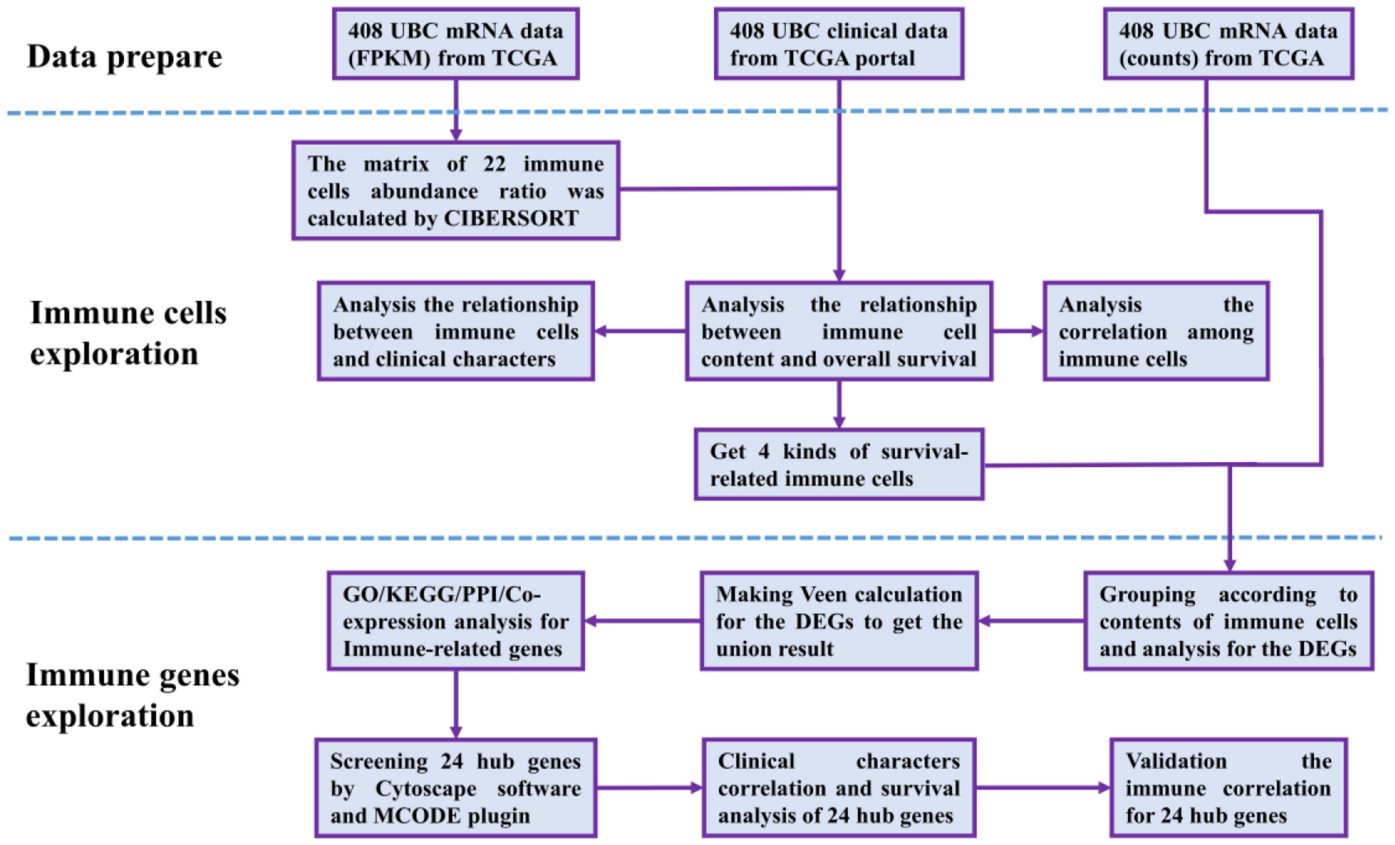
Flow chart of data processing in this study. TCGA, The Cancer Genome Atlas (https://portal.gdc.cancer.gov/). FPKM and counts are the two different mRNA data formats in the TCGA database. CIBERSORT is a web tool to estimate the abundance ratios of member cell types in a mixed cell population, using gene expression data. DEGs, differentially expressed genes; GO, Gene Ontology; KEGG, Kyoto Encyclopedia of Genes, and Genomes; PPI, protein-protein interactions. Cytoscape is a network processing software, and MCODE is a plugin in Cytoscape.

The RNA-Seq gene expression profiles of patients with BUC, including the FPKM and count format, were downloaded from the TCGA database using the *gdc-client* download tool. Clinical data, such as gender, age, tumor grade, clinical stage, and survival time, were also downloaded from the TCGA portal. Then, R software (R Foundation for Statistical Computing, Vienna, Austria) was used for data extraction and sorting to obtain the gene expression matrices and clinical data. Subsequent analyses were conducted, and all analytical processes are shown in Figure 1.

### Identifying Survival-Related Immune Cells

CIBERSORT is an analytical tool developed by Newman to provide an estimate of the abundance ratio of member cell types in a mixed cell population using gene expression data (14). We ran CIBERSORT locally in R software (15). The RNA-Seq (FPKM format) of BUC was analyzed to obtain the abundance ratio matrix of 22 immune cells. In total, 218 samples were selected with *P* ≤ 0.05. Then, a correlation analysis was conducted among the contents of the 22 immune cells in the 218 samples. Next, the Kaplan-Meier analysis for overall survival was proceeded based on the abundance ratio of 22 immune cells whose cut-off level was set at the median value with the aid of R software and the Log-Rank was utilized to test. We identified survival-related immune cells according to the results of the Kaplan-Meier survival analysis.

### Clinical Relationship With Survival-Related Immune Cells

The relationship between the abundance ratio of immune cells and tumor grade, clinical stage, stage T, and stage N was analyzed by combining the abundance ratio of immune cells and the clinical features in the 218 samples. Two variates used the independent sample *t*-test, while more variates used one-way ANOVA test. In this way, we could better understand the relevance of the clinical relationship with survival-related immune cells.

### Identifying Immune-Related Genes

According to the grouping in step 2.2 and the survival-related cells identified, we analyzed and obtained the genes related to each immune cell infiltration level. The differentially expressed genes were analyzed with the edgeR R package and the condition that |logFC| > 1.5 and *P* < 0.05. A Venn calculation and visualization were conducted via the online tool (http://bioinformatics.psb.ugent.be/webtools/Venn/) to obtain unique results for these genes.

### Enrichment Analysis of Immune-Related Genes

Gene Ontology (GO) and Kyoto Encyclopedia of Genes and Genomes (KEGG) enrichment analyses were used to annotate the structure, functions, and pathways of the genes. The DAVID website (https://david.ncifcrf.gov/) is one of the most authoritative enrichment tools (16). We used DAVID to analyse immune-related genes in the GO and KEGG pathways. Counts ≥ 4 and *P* < 0.05 were set as the enrichment cut-offs to screen meaningful enrichment results. Counts indicate the number of genes enriched in one GO/KEGG term. *P*-value is the judgment of significance of enrichment results. The enrichment results were visualized via the ggplot2 R package.

### Protein-Protein Interaction Network Construction, Hub Genes, and Modules Analysis

The 933 immune-related genes were imported into the STRING database (https://string-db.org/), a web tool used to explore protein-protein interactions, and the combined-score was set to ≥ 0.4 (17). The interaction network consisted of 829 nodes and 4,850 edges. This network was reconstructed via Cytoscape software and analyzed via the “molecular complex detection (MCODE)” plugin. In total, 29 modules were analyzed, including 24 *seed genes*, which were named hub genes. Then, we analyzed and obtained the 50 highest related genes to the 24 hub genes and the co-expression network of these genes via the cBioPortal online website (http://www.cbioportal.org/) (18, 19). For the co-expression analysis, the “TCGA provisional” dataset was selected and set as 7.5 in “Filter Neighbors by Alteration (%).” Lastly, we selected two modules with a gene number > 50 from MCODE and the co-expression network to perform GO/KEGG analysis via DAVID. The enrichment cut-off value was set to an adj. *P* < 0.05 and count ≥ 5.

### Relationship Between Clinical Characteristics and Hub Genes

The relationship between hub genes and clinical characteristics was analyzed and visualized by the “Weighted Correlation Network analysis (WGCNA)” package in R. The 218 patients were grouped and analyzed for overall survival according to the expression level of the 24 hub genes, as for the Kaplan-Meier survival analysis of the immune cells.

### Validation of the Immune Correlation

For validating the immune correlation of 24 hub genes, we employed the method of Pearson correlation analysis to analyse the correlation between these hub genes and 22 immune cells, which have got via the CIBERSORT in section identifying survival-related immune cells. The correlation index r and corresponding *p*-value are visualized via canvasXpress R package. TIMER (https://cistrome.shinyapps.io/timer/) is a comprehensive resource to systematically analyse immune infiltrates across diverse cancer types. The abundances of six immune cells (B cells, CD4^+^ T cells, CD8^+^ T cells, neutrophils, macrophages, and dendritic cells [DCs]) were estimated by a special statistical method, which was validated using a pathological estimate and the results are reliable (20). Survival-related hub genes were also validated for immune correlation via TIMER.

## Results

### Data Source and Pre-processing

The 408 cases of BUC data were downloaded and extracted into three matrices, including the RNA-Seq (FPKM and counts format) and clinical data. The subsequent analytical pre-processing for the study is shown in Figure 1.

### Identifying Survival-Related Immune Cells

**Figure 2:**
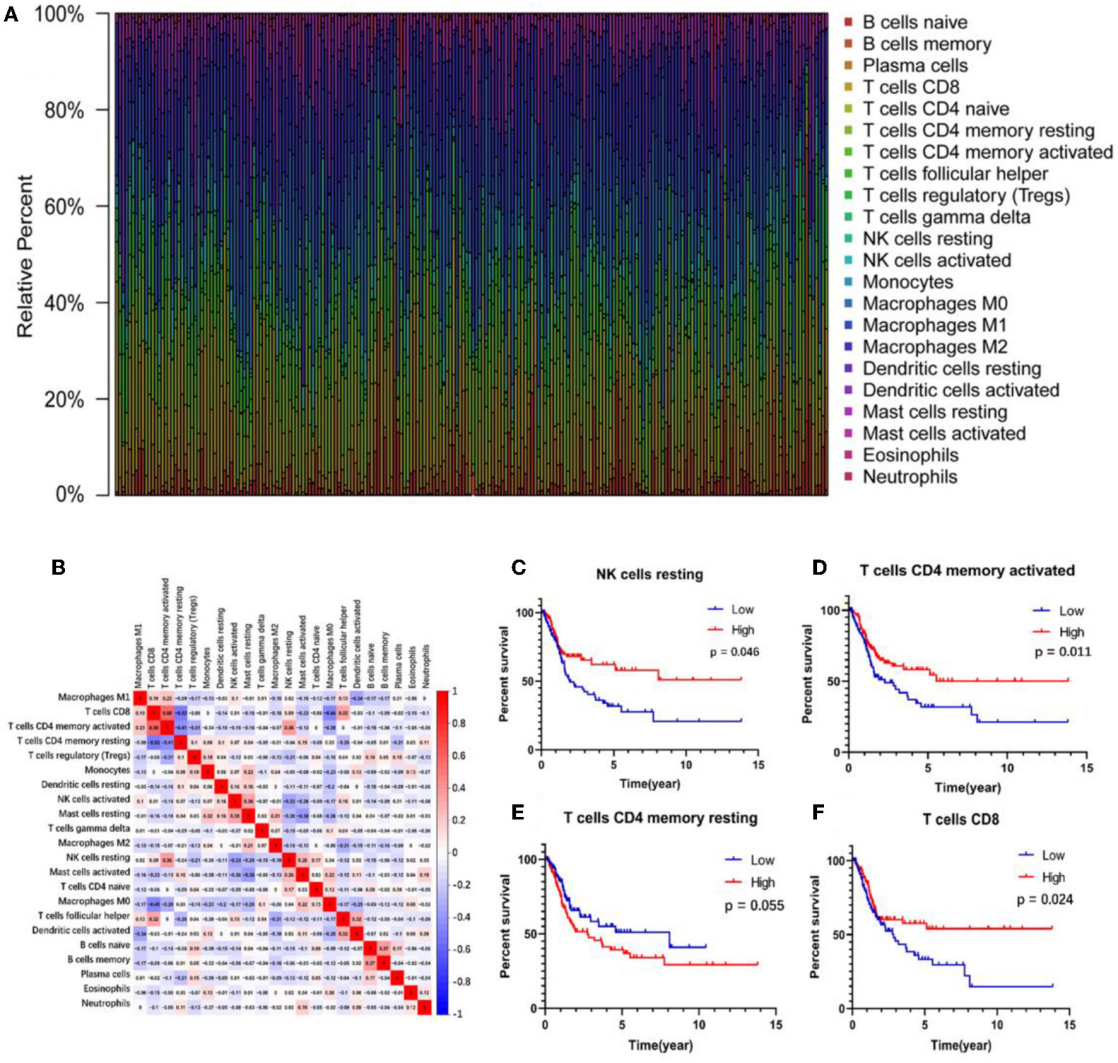
The relationship between the abundance ratios of immune cells and overall survival. **(A)** The abundance ratio of immune cells in the 218 samples. Each column represents a sample, and each column with a different color and height indicates the abundance ratios of immune cells in this sample. **(B)** The relationship between the abundance ratios of various immune cells. The value represents the correlation value. Red represents a positive correlation, and the blue represents a negative correlation. **(C–F)** The survival analysis for the abundance ratios of the four immune cells. The red line indicates a high expressing group of immune cells, and the blue line indicates a low expressing group of immune cells.

The abundance ratio of 22 immune cells in the 218 samples and their correlations were analyzed and are shown in Figures 2A,B. T cell CD4 memory activated and T cell CD8 contents were significantly correlated, while T cell CD4 memory resting was negatively correlated with T cell CD8 and T cell CD4 memory activated. Additionally, we analyzed the relationship between the abundance ratio of the 22 types of immune cells and overall survival via Kaplan-Meier analysis. The results in Figures 2C–F show that the abundance ratio of the four immune cells was related to survival, including T cell CD4 memory activated, T cell CD8, T cell CD4 memory resting, and natural killer (NK) cell resting.

### Clinical Relationship With Survival-Related Immune Cells

**Figure 3:**
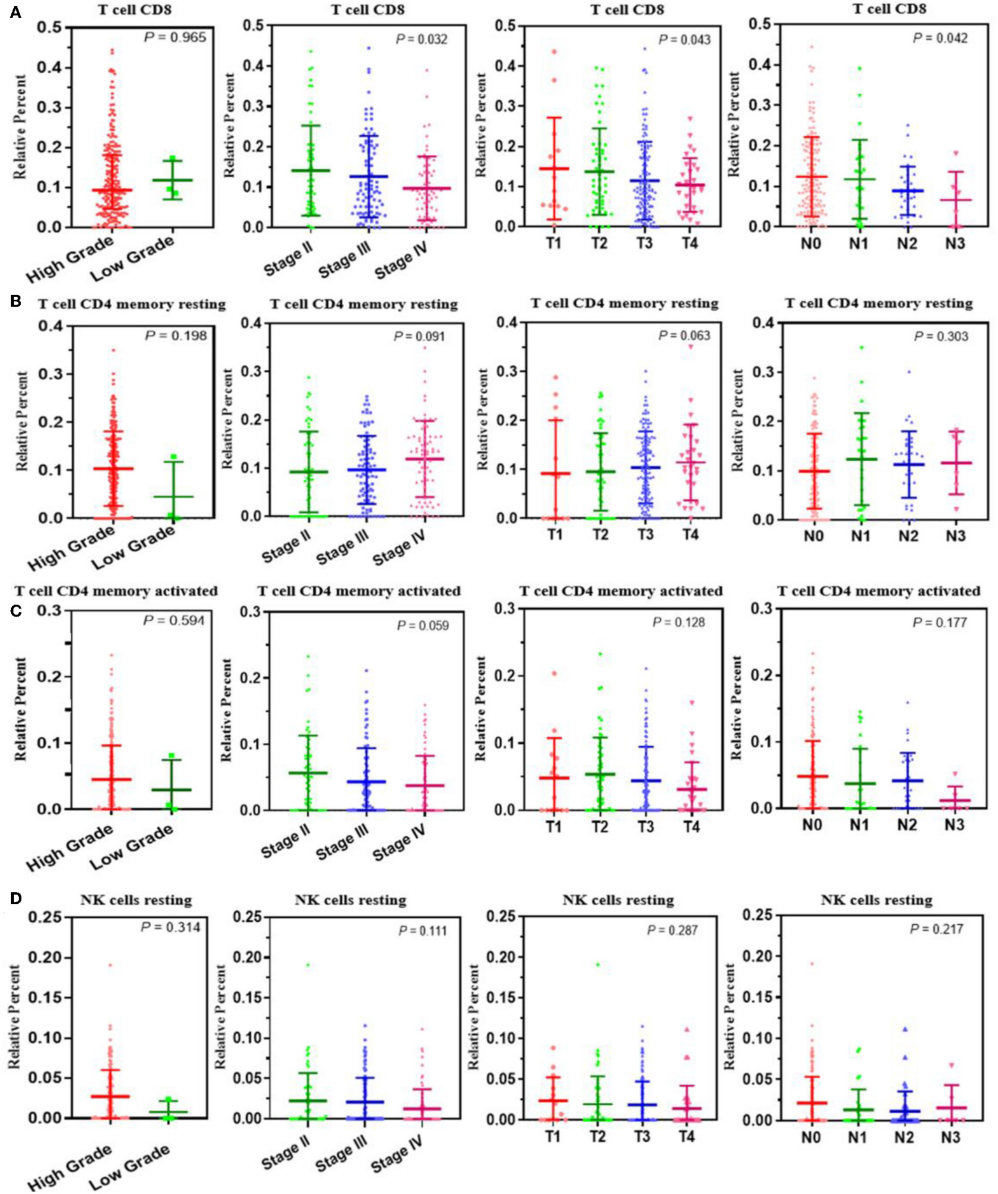
The relationship between the abundance ratios of the immune cells and clinical characteristics. **(A–D)** The relationship between the abundance ratios of each immune cell and tumor grade, clinical stage, T stage, and N stage. Each dot signifies the abundance ratio of an immune cell in a sample. The three horizontal line in each picture means mean ± SD.

A correlation analysis was carried out between the contents of the four survival-related immune cells and the clinical characteristics (including stage T, stage N, clinical stage, and tumor grade) to determine the effect of the immune cell abundance ratio on BUC clinical features. As shown in Figure 3, the abundance ratio of T cell CD8, NK cell resting, and T cell CD4 memory resting decreased with the increase of stage T/stage N/clinical stage, while the abundance ratio of T cell CD4 memory activated increased in an opposite manner. Because there were only three cases of low-grade BUC, the relationship between BUC grade and survival-related immune cells was unclear.

### Identifying Immune-Related Genes

**Figure 4:**
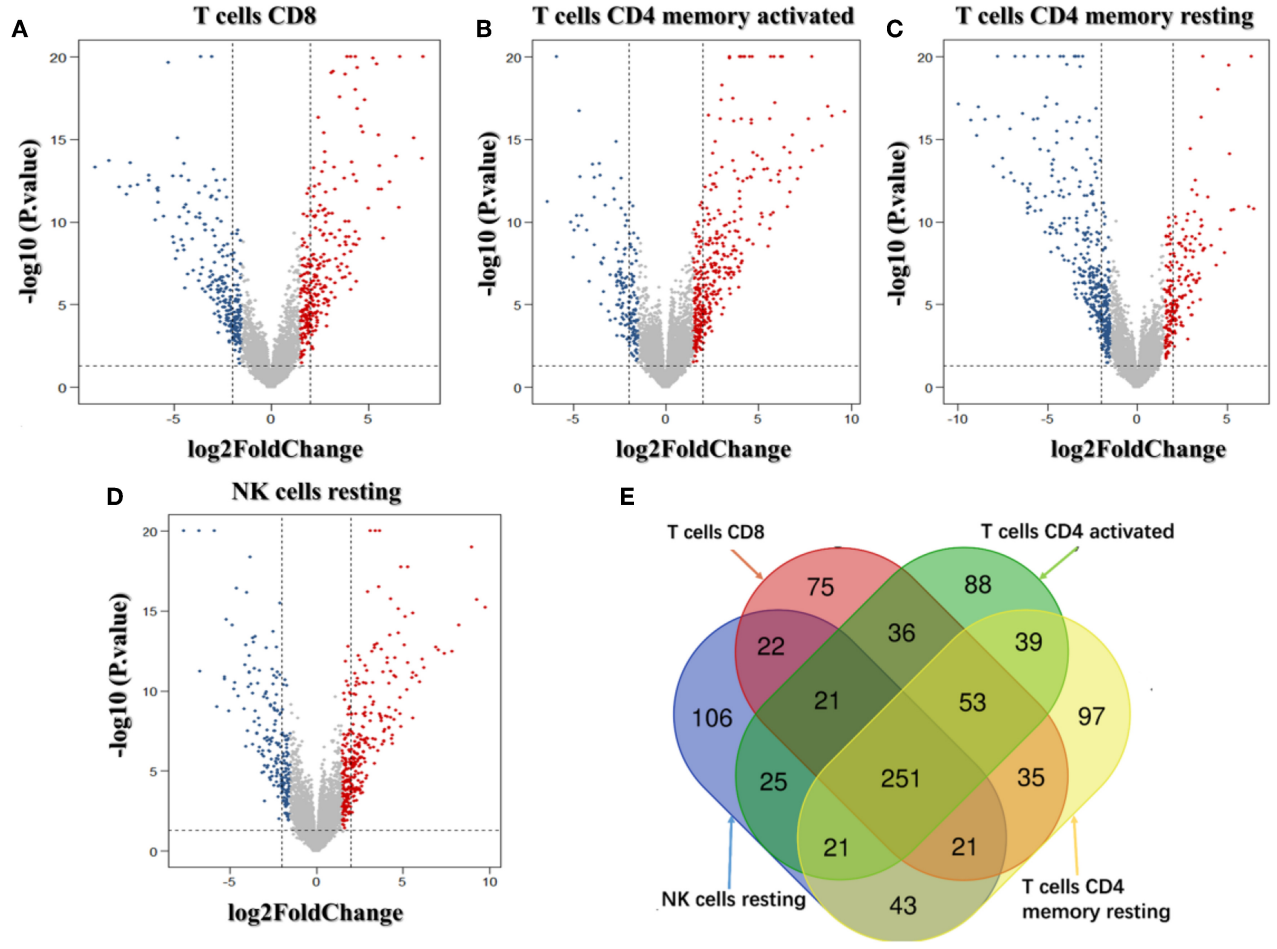
Identification of genes related to immune cell infiltration. **(A–D)** Volcano plots of the bladder urinary cancer gene expression profiles grouping by T cells CD8, T cells CD4 memory activated, T cells CD4 activated and NK cells resting. Red/blue symbols classify the upregulated/downregulated genes according to the criteria: |log2FC| > 1.5 and *P*-value < 0.05. **(E)** The Venn calculation result using the online tool (http://bioinformatics.psb.ugent.be/webtools/Venn/) to obtain genes involved in the infiltration of the four immune cells. The numbers in different color blocks represent the number of genes associated with immune cell infiltration. There are a total of 911 genes related to the infiltration of the four immune cells.

We analyzed the genes related to the levels of the four survival-related immune cells and found that 514 genes were related to T cell CD8, 510 to NK cell resting, 560 to T cell CD4 memory resting, and 534 to T cell CD4 memory activated. Volcano plots were used to show the results in Figures 4A–D. The Venn map analysis shown in Figure 4E revealed 933 genes totally related to immune cells infiltration.

### Enrichment Analysis of Immune-Related Genes

**Figure 5:**
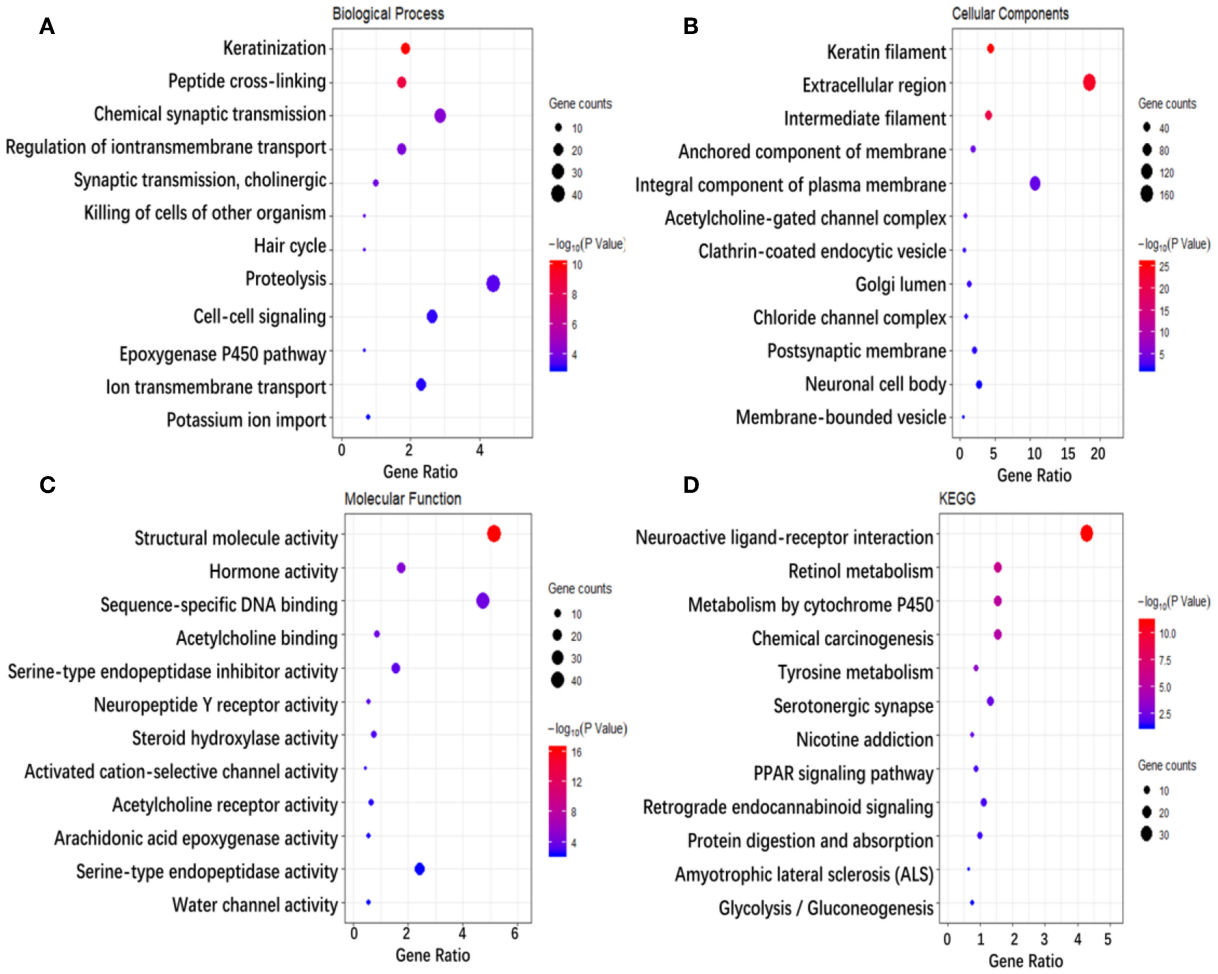
Enrichment analysis of genes related to immune cell infiltration. **(A–D)** Represent the enrichment analysis results of genes involved in immune cell infiltration, namely biological processes, cellular components, molecular functions, and KEGG. The main 12 results of each term are shown, and the color indicates the significant degree of enrichment and the size indicates the number of genes enriched for each result.

To investigate the biological classifications of immune-related genes, a GO/KEGG enrichment analysis was performed using the DAVID website, and the top 12 enrichment results for each term are plotted in Figure 5. GO analysis results showed that changes in the biological process (Figure 5A) of immune-related genes were significantly enriched in keratinization, peptide cross-linking, regulation of ion transmembrane transport, ion transmembrane transport, cell-cell signaling, etc. Changes in the molecular function (Figure 5B) were mainly enriched in keratin filament, extracellular region, intermediate filament, anchored component of membrane, Golgi lumen, acetylcholine-gated channel complex, etc. Changes in cellular component (Figure 5C) were mainly enriched in sequence-specific DNA binding, serine-type endopeptidase inhibitor activity, neuropeptide Y receptor activity, arachidonic acid epoxygenase activity, serotonin-activated cation-selective channel activity, etc. KEGG pathway analysis (Figure 5D) demonstrated that Metabolism of xenobiotics by cytochrome P450, Chemical carcinogenesis, Tyrosine metabolism, PPAR signaling pathway, etc. In brief, 933 immune-related genes were mainly involved in the transmission of various signaling pathways, as well as transportation and metabolism of nutrients.

### Protein-Protein Interaction Network Construction, Hub Genes, and Module Analysis

**Figure 6:**
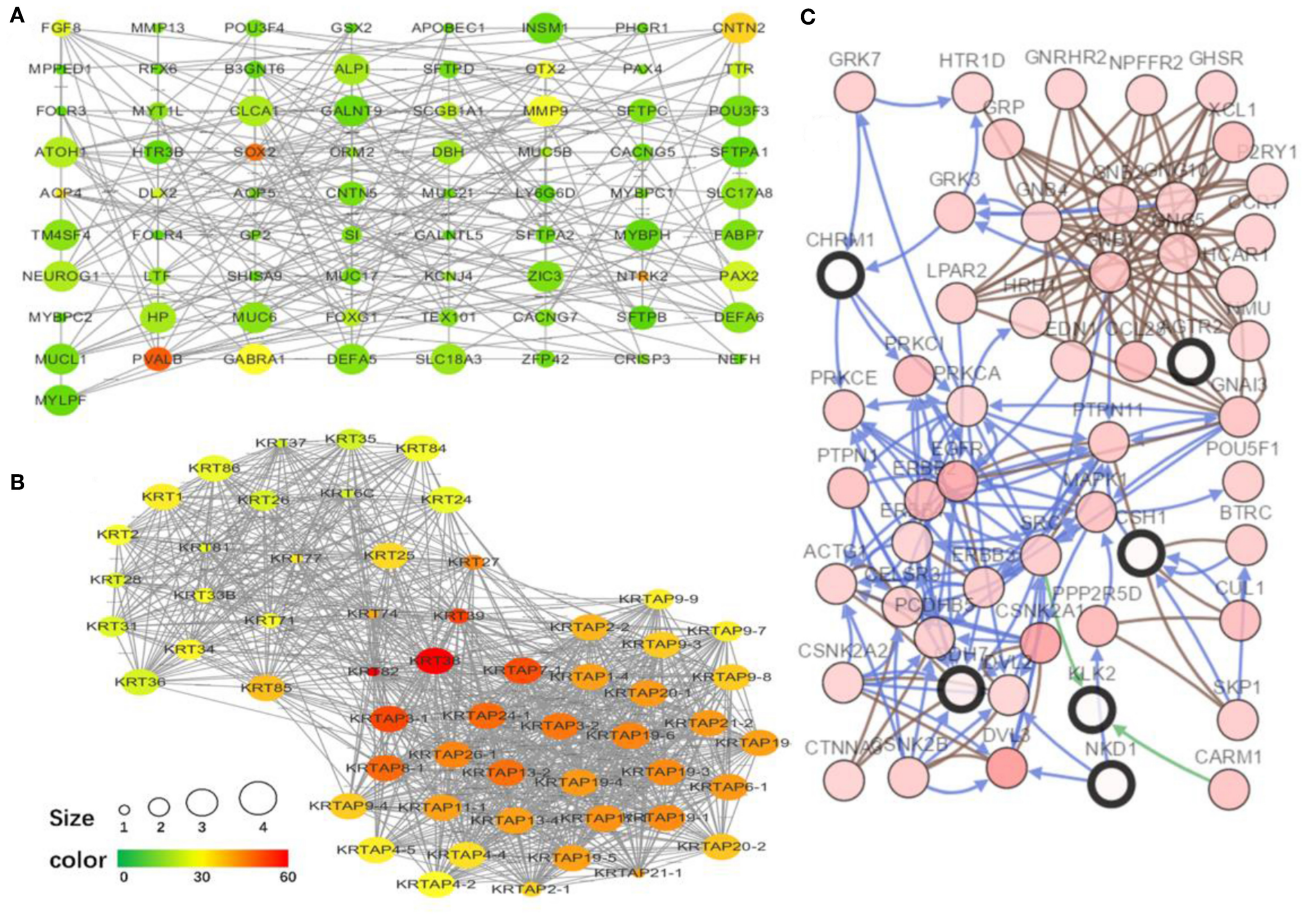
The top two modules and co-expression network. **(A,B)** Two modules with more than 50 genes in MCODE. The size indicates the number of immune cells associated with the gene, ranging from 1 to 4. The color indicates the number of proteins interacting with the other proteins. A redder color indicates a higher number, while green indicates a lower number. **(C)** The co-expression network of the 24 hub genes and 50 co-expressed genes. The figure was obtained using the online tool (http://www.cbioportal.org/). White and red represent the hub and co-expressing genes, respectively.

To explore the interrelation of immune-related genes and obtain hub genes, we made a PPI and module analysis, and obtained 24 hub genes, 2 modules with genes >50, 50 co-expression genes, and the co-expression network. The information of the 24 hub genes is shown in Table 1, including the full gene names and primary functions. The two modules with genes >50 and the co-expression networks are shown in Figures 6A–C, respectively. According to the results of the enrichment analysis for three modules shown in Table 2, module 1 genes were mainly related to protein transport and metabolism, module 2 genes were mainly related to the composition of keratin and intermediate filaments, and the co-expression network genes were mainly related to various signaling pathways associated with cancer. Of the three modules, the most interesting and important one is the co-expression network. It is involved in many pathways related to cancer and immunity, such as G-protein coupled receptor signaling pathway, Wnt signaling pathway, regulation of phosphatidylinositol 3-kinase signaling, PI3K-Akt signaling pathway, ErbB signaling pathway, etc.

**Table 1:** Functional roles of the 24 hub genes.

**No**.	**Gene**	**Full name**	**Function**
1	KRTAP19-6	Keratin associated protein 19-6	Developmental biology and keratinization
2	CHRM1	Cholinergic receptor muscarinic 1	Monoamine GPCRs and peptide ligand-binding receptors
3	AGTR2	Angiotensin II receptor type 2	Agents acting on the renin-angiotensin system pathway, pharmacodynamics and peptide ligand-binding receptors
4	SPRR2F	Small proline rich protein 2F	Cross-linked envelope protein of keratinocytes
5	GPR32	G protein-coupled receptor 32	Signaling by GPCR and G alpha (s) signaling events
6	UGT2B15	UDP glucuronosyltransferase family 2 member B15	Carbohydrate binding and glucuronosyltransferase activity
7	PSD2	Pleckstrin and Sec7 domain containing 2	Phospholipid binding and ARF guanyl-nucleotide exchange factor activity
8	MPPED1	Metallophosphoesterase domain containing 1	Hydrolase activity
9	STMN2	Stathmin 2	calcium-dependent protein binding and tubulin binding
10	CST4	Cystatin S	cysteine-type endopeptidase inhibitor activity
11	DGKK	Diacylglycerol kinase kappa	Glycerolipid metabolism and Signaling by GPCR
12	DMRTC2	DMRT like family C2	DNA-binding transcription factor activity and sequence-specific DNA binding
13	KRTAP2-3	Keratin associated protein 2–3	Developmental biology and keratinization
14	CSH1	Chorionic somatomammotropin hormone 1	Peptide ligand-binding receptors and Growth hormone receptor signaling
15	DSG4	Desmoglein 4	Developmental biology and keratinization
16	LIN28A	Lin-28 homolog A	Developmental biology and Wnt/hedgehog/notch
17	NKD1	NKD inhibitor of Wnt signaling pathway 1	Wnt/hedgehog/notch and wnt signaling pathway and pluripotency
18	KLK2	Kallikrein related peptidase 2	Agents acting on the renin-angiotensin system pathway, pharmacodynamics and signaling by Rho GTPases
19	FOXN4	Forkhead box N4	DNA-binding transcription factor activity and chromatin binding
20	UNC93A	Unc-93 homolog A	Toll-like receptor binding
21	LUZP1	Leucine zipper protein 1	chromosome 1p36 deletion syndrome
22	OTOG	Otogelin	Structural molecule activity and alpha-L-arabinofuranosidase activity
23	CDH7	Cadherin 7	ERK signaling and nanog in mammalian ESC pluripotency
24	TRIM51	Tripartite motif-containing 51	No data available

**Table 2:** GO and KEGG pathway enrichment analysis of the top 2 modules and co-expression network.

**Modules**		**Description**	***P*.adjust**	**Count**
Modules 1	BP terms	O-glycan processing	1.95E-04	7
		Cellular protein metabolic process	6.24E-04	8
		Respiratory gaseous exchange	0.0141	5
	CC terms	Lamellar body	1.47E-05	5
		Clathrin-coated endocytic vesicle	2.81E-04	5
		Extracellular region	0.00142	21
		Golgi lumen	0.001888	7
		Extracellular space	0.001995	19
Modules 2	CC terms	Intermediate filament	1.84E-60	35
		Keratin filament	2.16E-40	26
	MF terms	Structural molecule activity	1.61E-30	23
Co-expression	BP terms	Signal transduction	1.79E-06	22
		G-protein coupled receptor signaling pathway	5.32E-05	18
		Platelet activation	4.55E-04	8
		Wnt signaling pathway	9.23E-04	9
		Positive regulation of ERK1 and ERK2 cascade	0.007655	8
		Regulation of phosphatidylinositol 3-kinase signaling	0.01949	6
		Positive regulation of cytosolic calcium ion concentration	0.020014	7
	CC terms	Plasma membrane	3.47E-04	36
	MF terms	Protein kinase activity	0.016453	10
	KEGG pathway	Chemokine signaling pathway	6.32E-06	13
		Adherens junction	3.90E-05	9
		Wnt signaling pathway	6.02E-04	10
		Cholinergic synapse	0.001299	9
		Glutamatergic synapse	0.001592	9
		GABAergic synapse	0.002653	8
		Pathways in cancer	0.003451	14
		Morphine addiction	0.004209	8
		Circadian entrainment	0.005621	8
		Retrograde endocannabinoid signaling	0.008465	8
		Serotonergic synapse	0.015818	8
		PI3K-Akt signaling pathway	0.03281	12
		Dopaminergic synapse	0.040082	8
		ErbB signaling pathway	0.041677	7

### Relationship Between Clinical Characteristics and Hub Genes

**Figure 7:**
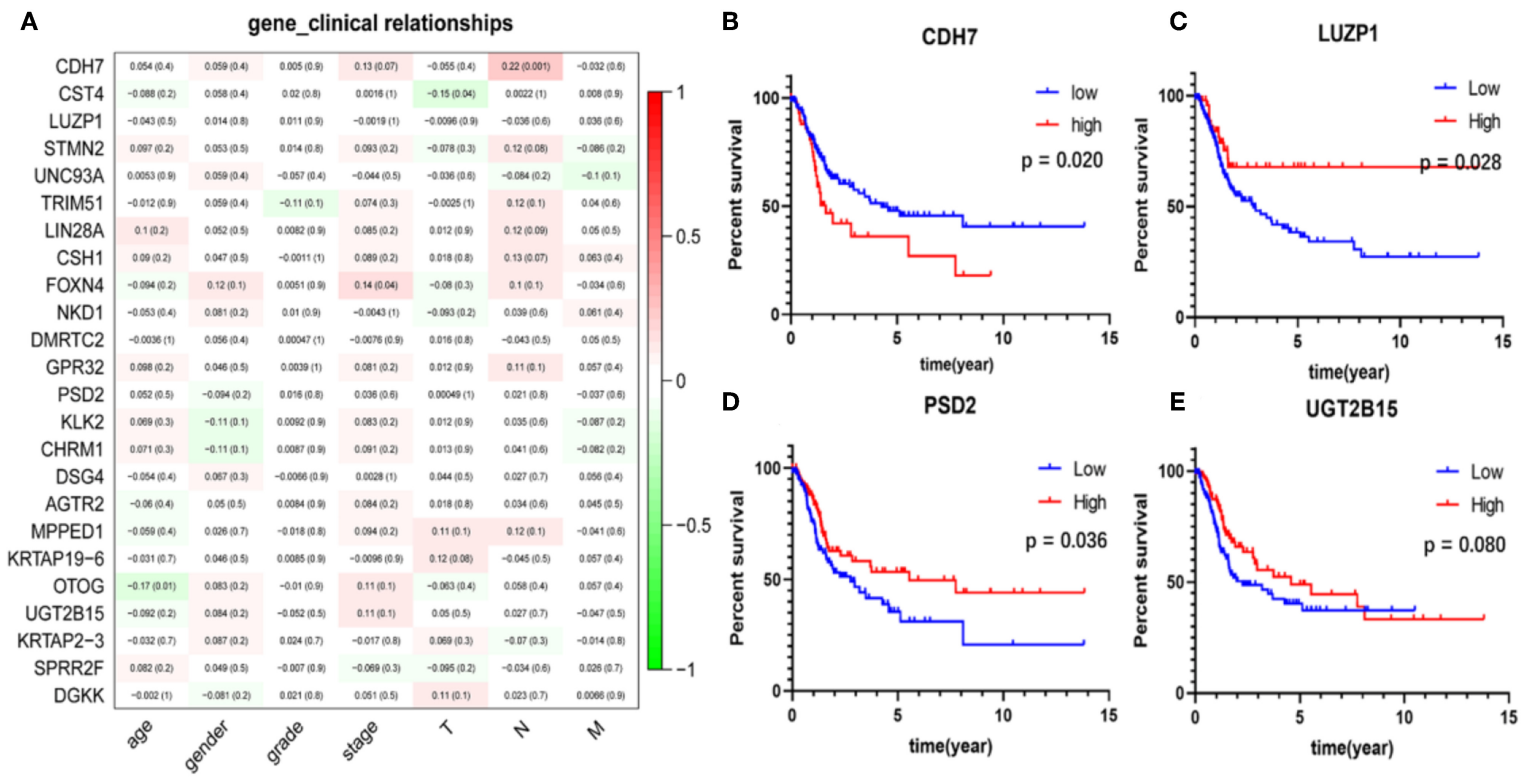
Clinical features correlation and survival analyses of the hub genes. **(A)** The correlation between the 24 hub genes and clinical characteristics. The former numbers in each small rectangle indicate the correlation and the numbers in brackets indicate the *P*-value for the correlation. **(B–E)** Are the four genes significantly related to survival, in which the red line indicates the group with higher expression of this gene, and the blue line indicates the group with lower expression.

The correlation analysis results of the clinical characteristics for the 24 hub genes are shown in Figure 7A. CDH7 was positively correlated with stage N, while CST4 was negatively correlated with stage T. The relationship between other hub genes and clinical characteristics can be easily found in the figure. The Kaplan-Meier survival analysis (Figures 7B–E) shows that four of the 24 hub genes strongly associated with clinical outcomes were CDH7, LUZP1, PSD2, and UGT2B15.

### Validation of the Immune Correlation

**Figure 8:**
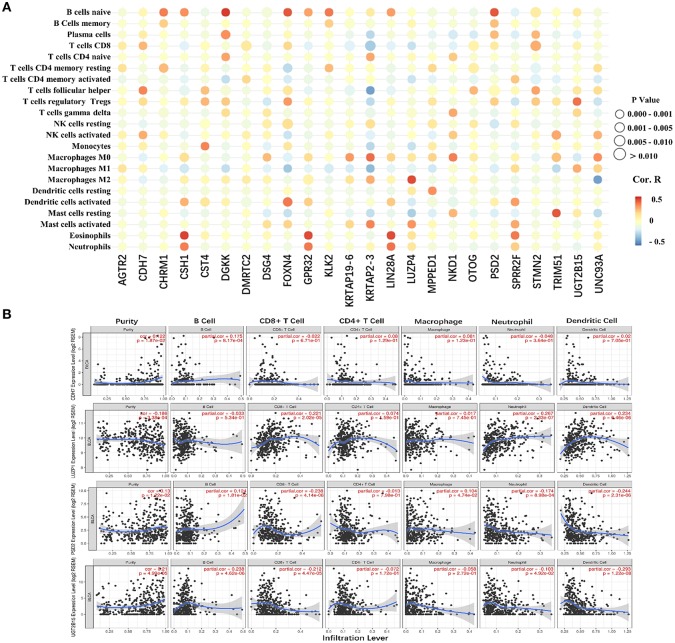
The correlation between the hub genes and various immune cells. **(A)** Red represents positive correlation genes and blue represents a negative correlation. The point size represents *P*-value and shade of color represents Pearson correlation index r. The x axis indicates hub genes and y axis indicates immune cell types. **(B)** Each dot represents a sample, and the blue line represents the relationship between the expression level of each gene and immune cell contents.

The 24 hub genes are potential immunotherapeutic targets, and their relationship and interaction with immune cells are of great value for farther immune-related research. The correlation analysis results between the 24 hub genes and 22 immune cells are shown in Figure 8A. TIMER was used to validate the correlation between the four survival-related genes and the level of immune cell infiltration, and the results are shown in Figure 8B. Part of hub genes, including four survival-related genes, were significantly correlated with some certain immune cell infiltrate.

## Discussion

Bladder cancer is the most common tumor of the urinary system, and its treatment progress has been slow. In recent years, the discovery of immune checkpoints has pushed cancer immunotherapy to a new level, achieving specific blockade of immunosuppressive effects and enhancing the anti-tumor immune response. Accumulating clinical data show that cancer immunotherapy is a key step in clinical cancer management (10). The purpose of our study was to screen and identify cells and genes closely related to immune infiltration and clinical outcomes in the BUC microenvironment. Our study not only identified cell and gene targets for BUC immunotherapy, but also proposes a new research idea for immunotherapy of other tumors.

In this study, four kinds of immune cells were related to the survival of patients with bladder cancer, including T cell CD8, T cell CD4 memory resting, T cell CD4 activated, and NK cell resting. CD8+ T cells are a hot spot in cancer research. Programmed death-1 (PD-1) on the surface of CD8+ T cells binds programmed death-ligand 1 (PD-L1) produced by tumor tissue, resulting in a limited host immune response. PD-L1 inhibitors increase the infiltration level of CD8+ T cells, which is an effective anti-tumor immune response (21). CD4+ memory T cells play an essential role in tumourigenesis and enlargement (22). CD4+ central memory T (TCM) cells maintain immune memory and exert immunoprotective effects during tumor metastasis (23, 24). CD4+ effector memory T (TEM) cells express adhesion molecules and chemokine receptors, which perform rapid functions (25). Both play a vital role in anti-tumor immunity, while TCM cells have more advantages than TEM cells (26). In the peripheral blood of patients with advanced cancer, the proportion of TCM cells decreases and TEM cells increases, presenting the typical T cell depletion status (27). In that study, patients with high T cell CD4 memory activated had shorter overall survival, while patients with high T cell CD4 memory resting had longer overall survival. This is consistent with the T cell depletion status theory. NK cells are an important part of the innate immune system, performing the function of memory antigen-specific immunity (28, 29). NK cells directly kill target cells, effectively remove diseased cells, and conduct immune surveillance. Some studies have shown that exercise-dependent mobilization of NK cells plays a crucial role in exercise-mediated protection against cancer (30–32). In summary, the four survival-related cells identified in this study are most likely to play an important role in immune infiltration as well as BUC immunotherapy, confirming that the analysis of immune-related genes based on the cells is credible.

The enrichment analysis of immune-related and co-expressed genes showed that these genes are mainly correlated with the transportation and metabolism of various nutrients (proteins, lipid, sugars, water, and ions) and various signaling pathways. By searching the signaling pathways enriched on the KEGG website (https://www.genome.jp/kegg/), we found that the PPAR signaling pathway, the epoxygenase P450 pathway, and the PI3K-Akt signaling pathway are involved in substance metabolism. The PI3K-Akt, Wnt, chemokine, and ErbB signaling pathways, as well as circadian rhythms and chemical carcinogenesis, are mainly associated with tumor cell metastasis, differentiation, survival, angiogenesis, and biological clocks. Metabolic change is an important feature of tumors. To meet the energy and biosynthetic demands of rapid proliferation, tumor cells use aerobic glycolysis for rapid energy supply (33). Different immune cell subsets also use different nutrients as an energy supply. Activated T cells, effector T (Teff) cells, including CD8+ T, CD4+ Th1, CD4+ Th2, and Th17, activated DCs, and activated M1 macrophages all use aerobic glycolysis, while the immunosuppressive cell subsets, such as regulatory T (Treg) cells, myeloid-derived suppressor cells, DC resting, and naive T cells use fatty acid oxidation to supply energy (34, 35). Thus, there is a competition between tumor cells and immune cells for energy. It has been shown that the severe nutrient deprivation in the tumor microenvironment allows Treg cells to use lactate as an energy substrate, while inhibiting lactate metabolism reduces Treg cell content (36). Similarly, promoting tryptophan degradation inhibits Teff cell function (37). These results indicate that the metabolic pattern of tumor tissue is related to immune cells, which can change the metabolism of specific substances to achieve different levels of immune cell infiltration and realize the treatment of tumors. Based on this evidence, we predict that these energy metabolism-related pathways act as an important bridge between immune infiltration and energy metabolism of BUC and are worthy of further study.

Of the enrichment analysis results, the PI3K-Akt signaling pathway is the clearest and important one to regulate immune environment. The general consensus is that the PI3K-Akt signaling pathway has the capacity to affect immune cell effector function and to regulate the immune-intrinsic features (38). Within the tumor microenvironment, a variety of immune cells co-exist in and interact with each other, and the activation of most immune cells are affected by the PI3K-AKT signaling pathway (39, 40). AKT can balance the terminal differentiation and production of memory CD8+T cells via regulating TCR, IL-2 receptor, and IL-12 receptor, etc. (41). Peng (42) and Abu-Eid et al. (43) found that PI3K inhibitor /AKT inhibitor could significantly increase the infiltration of CD8+T cells in tumor tissues and significantly prolong survival time. Meanwhile, AKT inhibitors can even effectively enhance the differentiation of other memory T cells in tumor tissues (44, 45). Abu-Eid et al. (43) found that PI3K-Akt pathway inhibitors selectively inhibit Tregs with minimal effect on conventional T cells to enhance the antitumour immune response. Okkenhaug et al. (46) proved that the PI3K-AKT-m TOR signaling pathway contributes to the development and activity of lymphocytes. Additional immune microenvironment features, such as the expression of immune checkpoint PD-L1 and inflammation within the tumor, are also modulated by the PI3K-AKT pathway (38). Other signaling pathways, such as Wnt, chemokine, chemical carcinogenesis, etc. are also more or less related to immunity.

Finally, a total of 24 hub genes were identified, four of which were related to survival, namely CDH7, LUZP1, PSD2, and UGT2B15. Previous studies have failed to investigate the relationship between these four genes and immunity, and only a few studies have shown that these genes are involved in the development of certain tumors. CDH7 is a typical adhesion molecule, and some studies have shown that CDH7 is a melanoma inhibitory protein binding partner that affects the migration of malignant melanoma cells (47–49). LUZP1 is a basic component of many proteins as well as a significant component of the group of membrane proteins on the surface of NK cells. Upregulation of LUZP1 is associated with a poor prognosis of liver cancer (50). UGT2B15 is associated with gastric cancer, breast cancer, and prostate cancer. UGT2B15 may upregulate FOXA1 and activate the hippocampal-yap signaling pathway to promote the development of gastric cancer (51). In breast cancer, UGT2B15 is regulated by sex hormone signaling in estrogen receptor-positive breast cancer (52). In human prostate cancer cells, androgen glucuronidation catalyzed by glucuronyltransferase is one of the main pathways to inactivate androgens, and high androgen expression is essential in the pathogenesis of prostate cancer (53).

In conclusion, four survival-related immune cells and 24 hub genes were identified, and four of these genes were shown to be related to overall survival in patients with BUC. These cells and genes can be considered biomarkers for prognosis, or as markers for bladder cancer therapy, which can be a focus of immunotherapy for bladder cancer. However, the evidence of this study remains indirect, and from bioinformatics, as with other similar studies. Through further research on these cells and genes, a new understanding of the potential relationship between the tumor microenvironment and BUC immunotherapy as well as prognosis can be achieved.

## Data Availability Statement

Publicly available datasets were analyzed in this study, these can be found in The Cancer Genome Atlas (https://portal.gdc.cancer.gov/).

## Author Contributions

The study conception and design were performed by JC and JT. Material preparation, data collection, and analysis were performed by JC, XY, JL, HW, PL, and ZY. The first draft of the manuscript was written by JC, JL, and ZD. All authors commented on previous versions of the manuscript. All authors read and approved the final manuscript.

### Conflict of Interest

The authors declare that the research was conducted in the absence of any commercial or financial relationships that could be construed as a potential conflict of interest.
